# Chemotherapy Associated Neutrophilic Eccrine Hidradenitis, an Unusual Case with Eccrine Squamous Syringometaplasia

**DOI:** 10.7759/cureus.6635

**Published:** 2020-01-12

**Authors:** Chantal Patel, Elizabeth Jones, Vivek Mudaliar, Manju Paul, Amir Ismail

**Affiliations:** 1 Surgery, University Hospitals of North Midlands, Newcastle-under-Lyme, GBR; 2 Dermatopathology, University Hospitals of North Midlands, Newcastle-under-Lyme, GBR; 3 Dermatology, University Hospitals of North Midlands, Newcastle-under-Lyme, GBR; 4 Plastic Surgery, University Hospitals of North Midlands, Newcastle-under-Lyme, GBR

**Keywords:** neutrophilic eccrine hidradenitis, chemotherapy, acute myeloid leukemia, eccrine squamous syringometaplasia

## Abstract

Neutrophilic eccrine hidradenitis (NEH) is a rare benign dermatological condition affecting the eccrine glands. The condition often occurs in response to chemotherapeutic agents in patients with acute myeloid leukemia (AML). However, cases of NEH are reported in patients with other malignancies and in those with non-malignant conditions. NEH is thought to result from the infiltration of neutrophils into the eccrine glands, resulting in erythematous papules and plaques on the skin. NEH is self-limiting and may resolve with cessation of the causative agent but can be treated symptomatically with steroids and analgesia. We report a case of NEH in a 52-year-old AML patient following the first cycle of chemotherapy. Following diagnosis, the patient was treated with a topical steroid and there was no recurrence. Alongside this, we uniquely present both clinical and histological images.

## Introduction

Neutrophilic eccrine hidradenitis (NEH) is a rare benign dermatological condition observed in patients with acute myeloid leukemia (AML). NEH commonly develops approximately 10 days after starting chemotherapy and often subsides soon after [1]. The areas commonly affected include the periorbital region, limbs and trunk [2]. NEH was originally described as a smooth lesion involving erythematous plaques and papules centimeters in size [3]. Its presentation is often confused with that of Sweet’s syndrome, another neutrophilic dermatosis presenting with erythematous pseudovesicular papules, nodules and plaques [4]. 

## Case presentation

**Figure 1:**
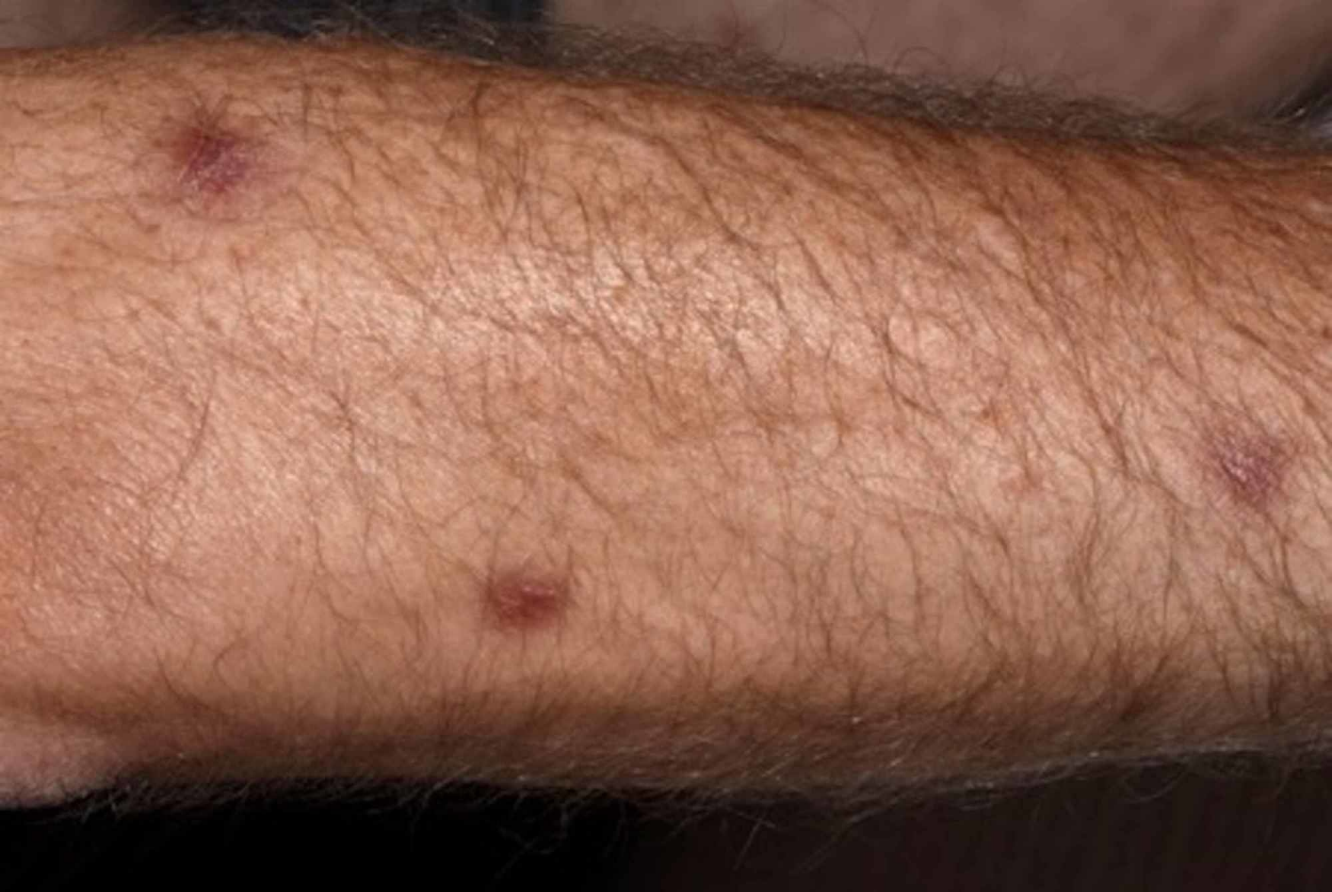
Solitary papules identified on the forearm of our patient

**Figure 2:**
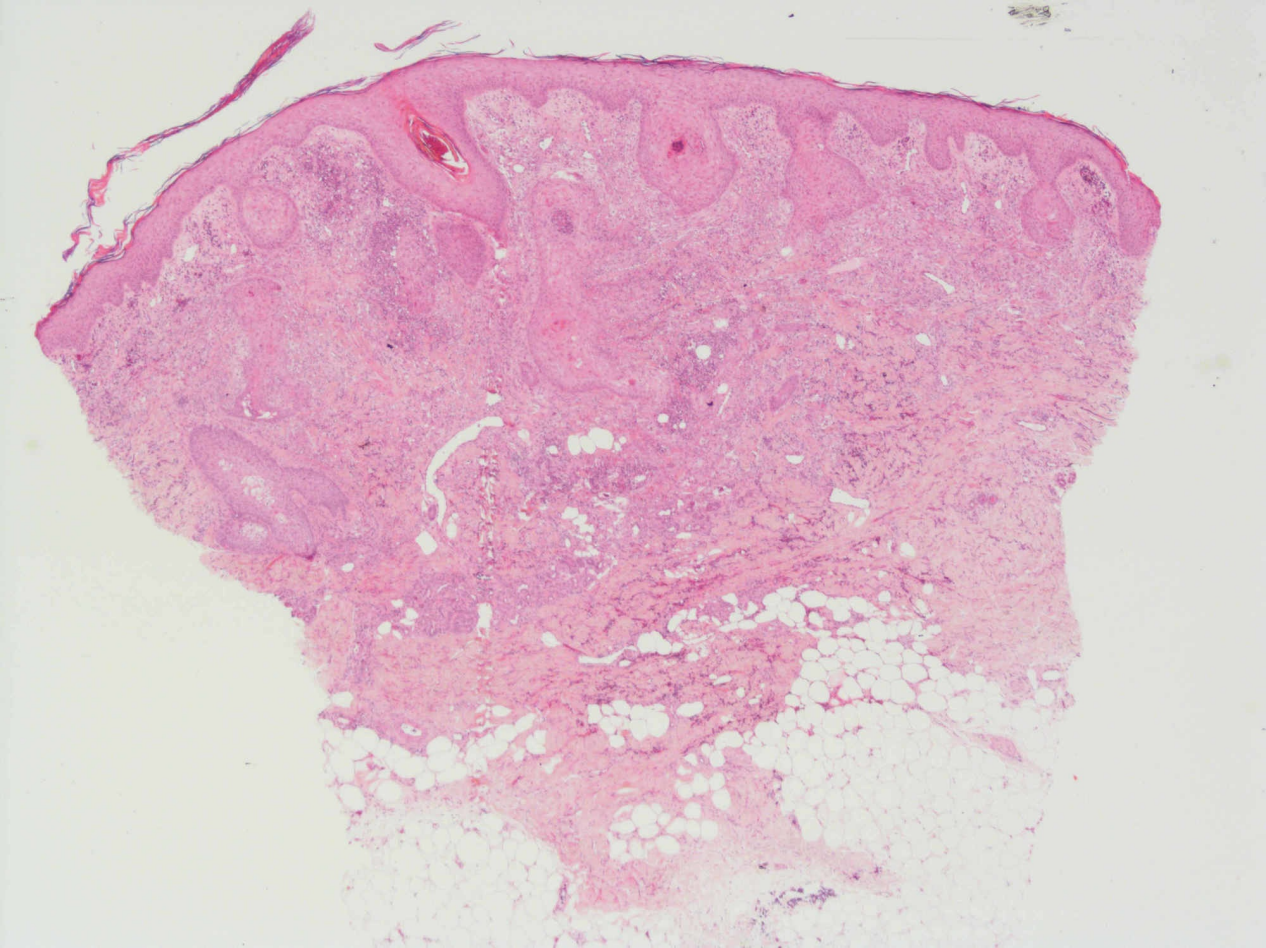
Biopsy revealing dermal neutrophilic infiltrate forming microabscesses (H&E, x4)

**Figure 3:**
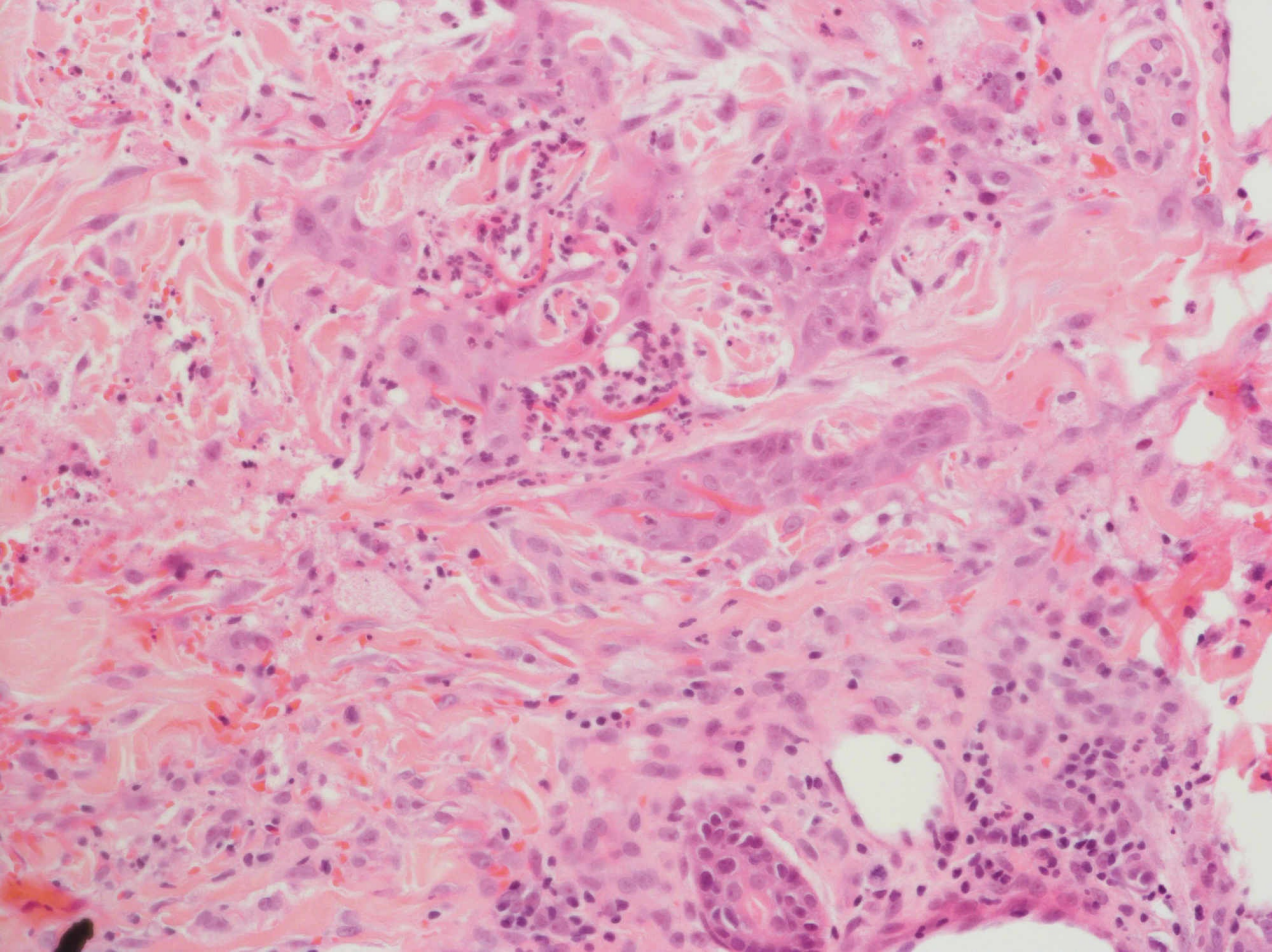
Biopsy revealing involvement of eccrine apparatus by the infiltrate on a high-power view (H&E, x20)

Our patient is a 52-year-old male, previously fit and well with a new diagnosis of primary AML. Following the first cycle of chemotherapy with cytarabine and daunorubicin, the patient developed asymptomatic juicy erythematous papules on the neck, trunk and limbs (Figure 1). Histology revealed pseudoepitheliomatous hyperplasia of the epidermis and prominent neutrophilic dermal infiltrates forming occasional microabscesses (Figure 2). The abscesses appeared to be associated with the epidermal downgrowths, with strands of mitotically active atypical squamous epithelium. Superficially this resembled squamous carcinoma. On a high-power view, these strands were frequently attached to or formed part of eccrine coils or ducts, and occasionally cuticles were seen (Figure 3). The appearances fall into the NEH/eccrine squamous syringometaplasia (ESS) spectrum. Mometasone furoate 0.1% was prescribed, and the patient continued on chemotherapy consisting of fludarabine, cytarabine, granulocyte-colony stimulating factor (G-CSF) and idarubicin. No further skin lesions were reported.

## Discussion

Approximately 90% of the cases of NEH were reported in oncology patients, 70% following their first cycle of chemotherapy [2,4]. A tentative link has been made with chemotherapeutic agents, most notably cytarabine [5]. However, identifying the specific causative agent in cases of NEH is often futile as combination chemotherapy is regularly prescribed [2].

The pathophysiology of NEH is yet to be defined. Metabolites derived from chemotherapeutic agents are thought to directly impact sweat glands causing necrosis of eccrine coil epithelium, subsequently leading to neutrophilic infiltration [3]. Other theories postulate NEH as a manifestation of paraneoplastic syndrome, belonging to a spectrum of neutrophilic dermatoses [3,4].

Cytarabine is commonly implicated in NEH, however, the pathophysiology is unknown [5]. NEH secondary to vasculitis due to the toxic effects of cytarabine has been argued [6]. Several cases describe a link between cytarabine and G-CSF. G-CSF is thought to perpetuate NEH by increasing chemotaxis and neutrophilic proliferation [1]. However, the resolution of NEH following the continuation of G-CSF has also been reported [7].

NEH is associated with ESS, and the two conditions are similar histologically [8]. In ESS, metaplasia of cuboidal cells to squamous cells occurs, often in response to chemotherapy. ESS can give the appearance of squamous cell carcinoma [9]. NEH and ESS can be grouped under the term ‘toxic erythema of chemotherapy’; both conditions are associated with cytarabine [5,8].

NEH recurrence has been reported [4]. Treatment is symptomatic; analgesia and corticosteroids can be prescribed [5]. The use of dapsone as a preventative measure prior to chemotherapy may prevent recurrence [10].

## Conclusions

Our case regarding NEH highlights the incidence of skin changes following chemotherapy and raises the importance of informing patients of adverse reactions following treatment with chemotherapeutic agents to allow for prompt management. NEH is a rare condition with an association with ESS and should be included in differential diagnoses for patients presenting with skin lesions post-chemotherapy. However, clarification of the underlying disease pathophysiology is yet to be achieved. 
